# Six-Month Incidence and Persistence of Oral HPV Infection in HIV-Negative and HIV-Infected Men Who Have Sex with Men

**DOI:** 10.1371/journal.pone.0098955

**Published:** 2014-06-04

**Authors:** Sofie H. Mooij, Hein J. Boot, Arjen G. C. L. Speksnijder, Chris J. L. M. Meijer, Audrey J. King, Dominique W. M. Verhagen, Henry J. C. de Vries, Wim G. V. Quint, Anco Molijn, Maurits N. C. de Koning, Marianne A. B. van der Sande, Maarten F. Schim van der Loeff

**Affiliations:** 1 Cluster of Infectious Diseases, Public Health Service of Amsterdam, Amsterdam, the Netherlands; 2 Centre for Infectious Disease Control, National Institute for Public Health and the Environment (RIVM), Bilthoven, the Netherlands; 3 Department of Pathology, Vrije Universiteit-University Medical Center, Amsterdam, the Netherlands; 4 Department of Internal Medicine, Jan van Goyen Medical Center, Amsterdam, the Netherlands; 5 Center for Infection and Immunity Amsterdam (CINIMA), Academic Medical Center, University of Amsterdam, Amsterdam, the Netherlands; 6 Department of Dermatology, Academic Medical Center, University of Amsterdam, Amsterdam, the Netherlands; 7 DDL Diagnostic Laboratory, Rijswijk, the Netherlands; 8 Julius Center for Health Sciences and Primary Care, University Medical Center Utrecht, Utrecht, the Netherlands; Georgetown University, United States of America

## Abstract

**Objectives:**

Our aim was to assess incidence and persistence of oral HPV infection in HIV-negative and HIV-infected men who have sex with men (MSM).

**Methods:**

MSM aged ≥18 years were included in Amsterdam (the Netherlands) in 2010–2011, and followed up 6 months later. Participants completed risk factor questionnaires. HPV DNA was analyzed in oral-rinse and gargle specimens using the SPF_10_-PCR DEIA/LiPA_25_ system (version 1). A subset of oral samples was subjected to SPF_10_ sequencing to identify additional HPV types. Multivariable logistic regression analyses using generalized estimating equations (GEE) were performed to assess determinants for oral high-risk HPV incidence and persistence.

**Results:**

689/795 participant MSM provided both baseline and 6-month data. Baseline prevalence of high-risk HPV was 9.4% in HIV-negative and 23.9% in HIV-infected MSM (*P*<0.001). 56/689 MSM acquired ≥1 high-risk HPV infection (6-month incidence 8.1%; 95%CI 6.2–10.4%); incidence was 4.1% in HIV-negative and 14.1% in HIV-infected MSM (*P*<0.001). HIV infection and recent use of cannabis were both independently associated with high-risk HPV incidence. Persistent high-risk HPV was observed in 48/130 (36.9%) infections.

**Conclusion:**

Incidence of oral high-risk HPV infection in MSM is substantial, and is associated with HIV infection. Over a third of HPV infections persisted over a 6-month period.

## Introduction

Oral infection with high-risk (oncogenic) Human Papillomavirus (HPV), in particular HPV type 16, is associated with a subset of head and neck cancers [1]–[4]. The overall HPV prevalence in head and neck squamous cell carcinomas is estimated to be 26% [3], with highest prevalence of 36–70% reported in oropharyngeal cancer [3], [5], [6]. While alcohol consumption and smoking have long been known as the main risk factors for head and neck cancer [7], emerging evidence also suggests HPV plays an important role with a rising incidence of HPV-related head and neck cancer in many parts of the world [6], [8], [9].

Oral HPV infection is thought to be transmitted sexually [10]–[13], and higher prevalence has been reported among HIV-infected individuals [10], [12]. Oral high-risk HPV prevalence in the general population is estimated to be around 3.5% [13], [14], but is much higher among HIV-infected men who have sex with men (MSM) [10], [15], who are also known to be at increased risk for HPV-related cancers [16], [17].

Two prospective studies among young, mostly heterosexual (male) adults indicated that sexual behavior, including kissing, may be associated with oral HPV acquisition [18], [19], and that most infections are likely to be transient rather than persistent [19]. Recently, a large study among men observed no independent association between oral sexual behavior and oral high-risk HPV incidence [20].

Improved insight into the natural history of oral HPV infection could have important public health implications, especially regarding possible options for prevention of HPV-related head and neck cancer, such as HPV vaccination. This study investigated the 6-month oral high-risk HPV incidence and persistence among MSM, focusing on the role of HIV infection, sexual behavior, and drug use.

## Materials and Methods

### Ethics statement

The Medical Ethics Committee of the Academic Medical Center (AMC) Amsterdam approved this study, and all participants provided written informed consent prior to participation.

### Study participants

The HIV & HPV in MSM (H2M) study design and sample collection has been described previously [15]. In brief, HIV-negative and HIV-infected MSM aged 18 years or older were recruited for a prospective cohort study in 2010–2011 at three sites in Amsterdam, the Netherlands: the Amsterdam Cohort Study (ACS) among MSM (Public Health Service of Amsterdam) [21], a Sexually Transmitted Infection (STI) clinic (Public Health Service of Amsterdam) [22], and an outpatient Infectious Disease clinic (Jan van Goyen Medical Center). Participants were followed up with every 3 to 6 months, for a maximum follow-up period of 24 months per person.

### Sample and data collection

At each visit, participants rinsed the oral cavity and gargled for 30 seconds, using 10 to 15 ml Scope mouthwash (Procter & Gamble, Toronto, Ontario). Participants completed self-administered questionnaires, with questions regarding health-related issues, drug use, and sexual behavior. Data pertaining to baseline and 6-month visits were included in the current analyses. HIV-related data were obtained at baseline from the national HIV patients' database of the Dutch HIV Monitoring Foundation.

### HPV DNA genotyping and classification

Preceding storage at −20°C, oral samples were washed and concentrated [15]. DNA extraction was performed using the MagNA Pure LC Total Nucleic Acid Isolation Kit (Roche, Mannheim, Germany), and subsequently HPV DNA amplification was performed using the highly sensitive SPF_10_-PCR DEIA/LiPA_25_ system (version 1) [23]. PCR products were tested for HPV DNA with a DNA Enzyme Immuno Assay (HPV DEIA, Labo Bio-medical Products, Rijswijk, the Netherlands). The DEIA uses general probes detecting a large spectrum of >68 HPV types. DEIA-positive samples were genotyped by a reverse-hybridization line probe assay (LiPA_25_) (HPV LiPA_25_, Labo Bio-medical Products, Rijswijk, the Netherlands). LiPA_25_ allows simultaneous identification of 25 specific mucosal HPV genotypes: 6, 11, 16, 18, 31, 33, 34, 35, 39, 40, 42, 43, 44, 45, 51, 52, 53, 54, 56, 58, 59, 66, 68/73, 70, and 74. The following HPV types were considered high-risk: 16, 18, 31, 33, 35, 39, 45, 51, 52, 56, 58, and 59 [1]. The remaining HPV types 6, 11, 34, 40, 42, 43, 44, 53, 54, 66, 68/73, 70, and 74 were classified as low-risk. Samples that were PCR-DEIA-positive and LiPA_25_-negative were classified as untypable.

In order to evaluate which HPV types other than the 25 types covered by LiPA_25_ were present in oral samples, a subset of oral samples was additionally subjected to SPF_10_ sequencing. The following samples were selected for sequencing: 1) baseline oral samples (n = 106) and 6-month oral samples (n = 119) classified as untypable, and 2) 6-month oral samples of participants with an untypable oral sample at baseline, regardless of the result of that 6-month sample (n = 67 additional samples). Sequencing was performed at DDL Diagnostic Laboratory (Rijswijk, the Netherlands).

A report on baseline prevalence of oral HPV and associations with baseline characteristics was published previously [15]; here we focus on the analysis of risk factors for oral high-risk HPV incidence and persistence.

### Statistical analyses

HIV-negative and HIV-infected participants were compared using rank sum tests for continuous data, and Chi-square tests for categorical data. Type-specific HPV incidence and persistence was estimated with exact 95% confidence intervals (95%CI). Univariable and multivariable logistic regression analyses using generalized estimated equations (GEE) were performed to assess associations between determinants and the 2 primary outcomes, i.e. oral high-risk HPV incidence and persistence. Logistic regression analysis was used rather than Poisson or proportional hazards analysis, because there were only 2 time points per participant, and there was limited variation in follow-up time. GEE was used to account for multiple HPV infections per person, whereby the unit of analysis was each individual high-risk HPV type. In a single GEE logistic regression model, each high-risk HPV type was treated as a separate end point, and then the exposure effect on high-risk HPV as a whole was estimated, taking correlations (i.e. multiple records per person) into account, assuming an exchangeable correlation structure [24].

We performed univariable and multivariable analyses to assess determinants for oral HPV incidence and persistence. The following variables were included a priori in each model: HPV type, HIV status, age, smoking pack years, and active oral-anal contact. In addition, we included variables that were associated with the outcome at *P*<0.1 in univariable analyses in HIV-negative or HIV-infected MSM. No backward selection was performed.

In case two variables were highly correlated (e.g., recent number of oral and anal sex partners), only one of these two variables was included in the model based on relevance or completeness of data. To minimize loss of observations in multivariable models, an extra category was created for each variable with ≥5% missing values. *P* values were 2-sided and considered statistically significant at *P*<0.05. All analyses were performed using Stata software package version 11.2 (Stata Intercooled, College Station, TX, USA).

## Results

### Participant characteristics

The current analysis encompasses 689 MSM (86.7%) who provided both baseline and 6-month oral samples and questionnaire data, of a total of 795 MSM from the H2M study. The excluded MSM (n = 106) were comparable to the included MSM with respect to age and HIV status. At enrollment, 276/689 (40.1%) MSM were HIV-infected. Baseline characteristics and time-updated behavioral data from the 6-month visit are shown in Table 1. The median age at baseline was 38 years for the 413 HIV-negative and 47 years for the 276 HIV-infected MSM (*P*<0.001). HIV-infected MSM reported more smoking pack years and recent use of cannabis. Also, HIV-infected MSM reported more recent and lifetime sexual partners compared to HIV-negative MSM.

**Table 1:** Characteristics of 689 MSM participating in the H2M study, overall and stratified by HIV status (Amsterdam, 2010–2012).

	Overall (n = 689)		HIV-negative MSM (n = 413)		HIV-infected MSM (n = 276)		*P* value^a^
	No.	%	No.	%	No.	%	
***A. Socio-demographic characteristics***							
**Median age in years (IQR)** c	40.3	(34.9–47.5)	37.5	(33.4–42.2)	46.5	(39.9–53.1)	**<0.001** b
**Age (years) by category** c							**<0.001**
≤34	174	25.3%	139	33.7%	35	12.7%	
35–44	306	44.4%	214	51.8%	92	33.3%	
≥45	209	30.3%	60	14.5%	149	54.0%	
**Country of birth** c							**0.04**
The Netherlands	559	81.8%	344	84.3%	215	78.2%	
Any other country	124	18.2%	64	15.7%	60	21.8%	
Missing values	6		5		1		
***B. Health-related characteristics***							
**Tobacco smoking pack years** c d							**0.001**
0–14	423	81.0%	283	85.2%	140	73.7%	
≥15	99	19.0%	49	14.8%	50	26.3%	
Missing values	167		81		86		
**Cannabis use last 6 months** e							**<0.001**
No	467	71.3%	306	76.9%	161	62.6%	
Yes	188	28.7%	92	23.1%	96	37.4%	
Missing values	34		15		19		
***C. Sexual behavior***							
**Median lifetime number of male sex partners (IQR)** c	200	(60–500)	100	(50–400)	300	(100–1000)	**<0.002** b
**Lifetime number of male sex partners by category** c							**<0.001**
≤100	267	41.6%	194	50.1%	73	28.6%	
101–500	216	33.6%	120	31.0%	96	37.6%	
≥501	159	24.8%	73	18.9%	86	33.7%	
Missing values	47		26			21	
**Anal sex last 6 months**							0.3
No	123	18.1%	69	16.9%	54	19.8%	
Yes	558	81.9%	339	83.1%	219	80.2%	
Missing values	8		5		3		
**Number of anal sex partners last 6 months by category**							**0.002**
≤1	307	45.3%	198	48.6%	109	40.2%	
2–4	167	24.6%	107	26.3%	60	22.1%	
≥5	204	30.1%	102	25.1%	102	37.6%	
Missing values	11		6		5		
**Number of oral sex partners last 6 months by category**							**0.05**
≤2	280	41.0%	162	39.3%	118	43.5%	
3–7	181	26.5%	123	29.9%	58	21.4%	
≥8	222	32.5%	127	30.8%	95	35.1%	
Missing values	6		1		5		
**Oral-anal contact last 6 months (rimming)**							0.08
No	255	38.1%	140	35.4%	115	42.0%	
Yes	415	61.9%	256	64.6%	159	58.0%	
Missing values	19		17		2		
***D. HIV-related characteristics***							
**Median CD4 cell count at enrollment (cells/mm^3^) (IQR)** c					530	(410–694)	
**Median nadir CD4 cell count (cells/mm^3^) (IQR)** c					220	(160–320)	
**HIV viral load at enrollment (copies/mL) by category** c							
<50					184	79.7%	
≥50					47	20.3%	
Missing values					45		
**Use of cART at enrollment** c							
No					29	12.2%	
Yes					209	87.8%	
Missing values					38		

Abbreviations: MSM  =  men who have sex with men; H2M  =  HIV & HPV in MSM; IQR  =  interquartile range; cART  =  combination antiretroviral therapy.

Significant results (*P*<0.05) are represented in bold font.

a Based on Chi-square test (except when stated otherwise).

b Based on rank sum test.

c As measured at enrollment. All other variables are based on questions asked during the 6-month visit.

d Participants who were not current smokers but provided no information on past smoking behavior were counted as missing.

Never smokers were included in category 0–14 pack years.

e This concerns cannabis use in general (without specifying route of administration).

Baseline prevalence of oral high-risk HPV infection was 9.4% (39/413) in HIV-negative and 23.9% (66/276) in HIV-infected MSM (*P*<0.001) (see earlier report on baseline data [15]). The median follow-up time between baseline and 6-month visit was 182 days (IQR 174–190) in HIV-negative and 174 days (IQR 126–207) in HIV-infected MSM (*P*<0.001).

### Six-month oral high-risk HPV incidence

**Figure 1:**
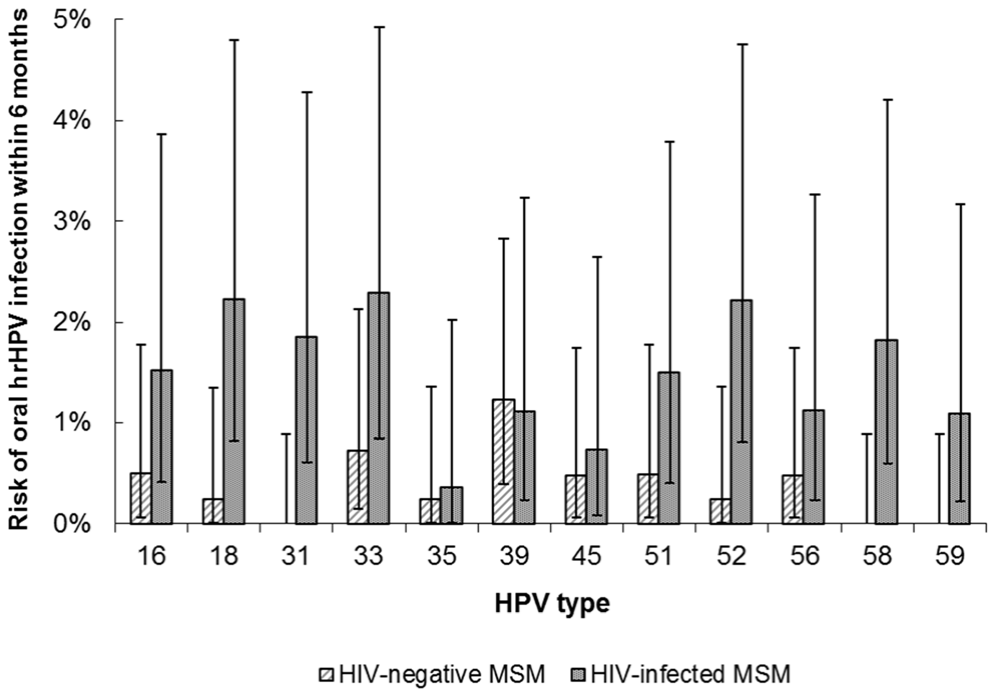
Type-specific 6-month oral high-risk HPV incidence. Type-specific 6-month oral high-risk HPV incidence and 95% confidence intervals in 413 HIV-negative and 276 HIV-infected MSM (H2M study, Amsterdam, 2010–2012). Abbreviations: MSM  =  men who have sex with men; H2M  =  HIV & HPV in MSM; hrHPV  =  high-risk HPV.

Incidence of ≥1 oral high-risk HPV infection at the 6-month visit was observed in 56/689 MSM (8.1%; 95%CI 6.2–10.4): incidence was significantly higher in HIV-infected MSM (39/276; 14.1%; 95%CI 10.2–18.8) than in HIV-negative MSM (17/413; 4.1%; 95%CI 2.4–6.5) (*P*<0.001). Type-specific 6-month incidence was higher in HIV-infected than in HIV-negative MSM for each HPV type, except for HPV-39 (Figure 1). In total, 67 incident high-risk HPV infections were observed, of which 19 infections occurred in HIV-negative MSM and 48 infections in HIV-infected MSM.

### Determinants for oral high-risk HPV incidence

HIV infection was significantly associated with oral high-risk HPV incidence in univariable analysis (odds ratio (OR) 3.9; 95%CI 2.2–7.0) (Table 2). In addition, recent use of cannabis, increasing number of lifetime male sex partners, and a higher number of recent oral sex partners were significantly associated with oral high-risk HPV incidence in univariable analysis. HIV infection was also significantly associated with oral high-risk HPV incidence in multivariable analyses (adjusted odds ratio (aOR) 3.3; 95%CI 1.7–6.3). Apart from HIV infection, recent use of cannabis remained borderline significantly associated (aOR 1.8; 95%CI 1.0–3.1; *P* = 0.05); a higher number of recent oral sex partners was also of borderline significance (overall *P* = 0.07). In an analysis restricted to HIV-infected MSM, neither detectable HIV viral load (aOR 0.5; 95% CI 0.1–1.5) nor CD4 cell count above 350 cells/mm^3^ at baseline (aOR 0.7; 95%CI 0.3–1.8) was associated with incidence.

**Table 2:** Determinants of 6-month oral high-risk HPV incidence in 689 HIV-negative and HIV-infected MSM participating in the H2M study (Amsterdam 2010–2012).

	Univariable analysis		Multivariable analysis	
	OR (95% CI)	*P* value	aOR (95% CI)	*P* value^a^
**HIV infection** b		**<0.001**		**<0.001**
No	1.0		1.0	
Yes	3.9 (2.2–7.0)		3.3 (1.7–6.3)	
**Age (years) by category** b		0.08		0.9
≤34	1.0		1.0	
35–44	1.4 (0.6–3.1)		1.3 (0.5–2.9)	
≥45	2.3 (1.0–4.9)		1.3 (0.5–3.0)	
**Tobacco smoking pack years** b c		0.07		0.2
0–14	1.0		1.0	
≥15	1.9 (0.9–3.7)		1.5 (0.7–3.1)	
**Cannabis use last 6 months** d		**0.001**		**0.05**
No	1.0		1.0	
Yes	2.5 (1.4–4.2)		1.8 (1.0–3.1)	
**Lifetime number of male sex partners** b		**0.04**		0.7
≤100	1.0		1.0	
101–500	1.6 (0.7–3.4)		0.7 (0.3–1.6)	
≥501	2.6 (1.2–5.4)		1.0 (0.4–2.2)	
**Number of oral sex partners last 6 months by category**		**0.006**		0.07
≤2	1.0		1.0	
3–7	1.0 (0.4–2.2)		1.1 (0.5–2.5)	
≥8	2.4 (1.3–4.5)		2.3 (1.0–4.9)	
**Oral-anal contact last 6 months (rimming)**		0.3		0.8
No	1.0		1.0	
Yes	1.4 (0.8–2.4)		1.1 (0.6–2.1)	

High-risk HPV: types 16, 18, 31, 33, 35, 39, 45, 51, 52, 56, 58, and 59. HPV type was included in the multivariable model, apart from the variables shown in the table.

Abbreviations: MSM  =  men who have sex with men; H2M  =  HIV & HPV in MSM; OR  =  odds ratio; aOR  =  adjusted odds ratio; 95% CI  =  95% confidence interval.

Significant results (*P*<0.05) are represented in bold font.

a Based on Wald test (excluding missing category).

b As measured at enrollment. All other variables are based on questions asked during the 6-month visit.

c Participants who were not current smokers but provided no information on past smoking behavior were counted as missing.

Never smokers were included in category 0–14 pack years.

d This concerns cannabis use in general (without specifying route of administration).

### Six-month oral high-risk HPV persistence

In total, 105 MSM were positive for ≥1 oral high-risk HPV infections at baseline; 6-month persistence was observed in 12/39 HIV-negative (30.8%) and 32/66 (48.5%) HIV-infected MSM (*P* = 0.08). Among these 105 MSM, a total of 130 baseline type-specific oral high-risk HPV infections were observed, of which 48 persisted (36.9%) and 82 were cleared (63.1%) after 6 months. Persistence was observed in 12/44 (27.3%) and 36/86 (41.9%) infections in HIV-negative and HIV-infected MSM, respectively (*P* = 0.1). Type-specific persistence, stratified by HIV status, is shown in Table 3.

**Table 3:** Type-specific 6-month oral high-risk HPV persistence with 95% confidence intervals in 413 HIV-negative and 276 HIV-infected MSM (H2M study, Amsterdam, 2010–2012).

	Overall (n = 689)		HIV-negative MSM (n = 413)		HIV-infected MSM (n = 276)	
	Positive at baseline	Persistent at 6 months (%; 95% CI)	Positive at baseline	Persistent at 6 months (%; 95% CI)	Positive at baseline	Persistent at 6 months (%; 95% CI)
**HPV-16**	23	12 (52.2%; 30.6–73.2)	9	3 (33.3%; 7.5–70.1)	14	9 (64.3%; 35.1–87.2)
**HPV-18**	10	3 (30.0%; 6.7–65.2)	3	0 (0.0%; 0.0–70.8)	7	3 (42.9%; 9.9–81.6)
**HPV-31**	8	2 (25.0%; 3.2–65.1)	1	0 (0.0%; 0.0–97.5)	7	2 (28.6%; 3.7–71.0)
**HPV-33**	18	13 (72.2%; 46.5–90.3)	4	3 (75.0%; 19.4–99.4)	14	10 (71.4%; 41.9–91.6)
**HPV-35**	9	5 (55.6%; 21.2–86.3)	6	4 (66.7%; 22.3–95.7)	3	1 (33.3%; 0.8–90.6)
**HPV-39**	12	1 (8.3%; 0.2–38.5)	4	0 (0.0%; 0.0–60.2)	8	1 (12.5%; 0.3–52.7)
**HPV-45**	6	0 (0.0%; 0.0–45.9)	1	0 (0.0%; 0.0–97.5)	5	0 (0.0%; 0.0–52.2)
**HPV-51**	17	4 (23.5%; 6.8–49.9)	8	1 (12.5%; 0.3–52.7)	9	3 (33.3%; 7.5–70.1)
**HPV-52**	9	0 (0.0%; 0.0–33.6)	4	0 (0.0%; 0.0–60.2)	5	0 (0.0%; 0.0–52.2)
**HPV-56**	12	6 (50.0%; 21.1–78.9)	2	1 (50.0%; 1.3–98.7)	10	5 (50.0%; 18.7–81.3)
**HPV-58**	2	1 (50.0%; 1.3–98.7)	0	NA	2	1 (50.0%; 1.3–98.7)
**HPV-59**	4	1 (25.0%; 0.6–80.6)	2	0 (0.0%; 0.0–84.2)	2	1 (50.0%; 1.3–98.7)
**Total**	130	48 (36.9%)	44	12 (27.3%)	86	36 (41.9%)

Abbreviations: MSM  =  men who have sex with men; H2M  =  HIV & HPV in MSM; 95% CI  =  95% confidence interval.

### Determinants for oral high-risk HPV persistence

In univariable analysis, HIV infection was not significantly associated with oral high-risk HPV persistence (OR 1.9; 95%CI 0.9–4.4) (Table 4). In multivariable analyses there were no determinants significantly associated with oral high-risk HPV persistence. In an analysis restricted to HIV-infected MSM, neither detectable HIV viral load (aOR 1.2; 95%CI 0.3–5.3) nor CD4 cell count above 350 cells/mm^3^ at baseline (aOR 0.6; 95%CI 0.1–2.4) was associated with persistence.

**Table 4:** Determinants of 6-month oral high-risk HPV persistence of 130 baseline infections in HIV-negative and HIV-infected MSM participating in the H2M study (Amsterdam 2010–2012).

	Univariable analysis		Multivariable analysis	
	OR (95% CI)	*P* value	aOR (95% CI)	*P* value^a^
**HIV infection** b		0.1		0.6
No	1.0		1.0	
Yes	1.9 (0.9–4.4)		1.3 (0.5–3.2)	
**Age (years) by category** b		0.06		0.2
≤34	1.0		1.0	
35–44	0.8 (0.2–3.7)		0.8 (0.2–3.5)	
≥45	2.1 (0.5–9.1)		1.7 (0.4–8.1)	
**Tobacco smoking pack years** b c		0.9		0.6
0–14	1.0		1.0	
≥15	1.1 (0.4–2.7)		0.8 (0.3–2.1)	
**Oral-anal contact last 6 months (rimming)**		0.1		0.4
No	1.0		1.0	
Yes	0.5 (0.2–1.2)		0.7 (0.3–1.6)	

High-risk HPV: types 16, 18, 31, 33, 35, 39, 45, 51, 52, 56, 58, and 59.

HPV type could not be included in the multivariable model, due to limited number of observations.

Abbreviations: MSM  =  men who have sex with men; H2M  =  HIV & HPV in MSM; OR  =  odds ratio; aOR  =  adjusted odds ratio; 95% CI  =  95% confidence interval.

Significant results (*P*<0.05) are represented in bold font.

a Based on Wald test (excluding missing category).

b As measured at enrollment. All other variables are based on questions asked during the 6-month visit.

c Participants who were not current smokers but provided no information on past smoking behavior were counted as missing. Never smokers were included in category 0- 14 pack years.

### Six-month oral low-risk HPV incidence and persistence

**Figure 2:**
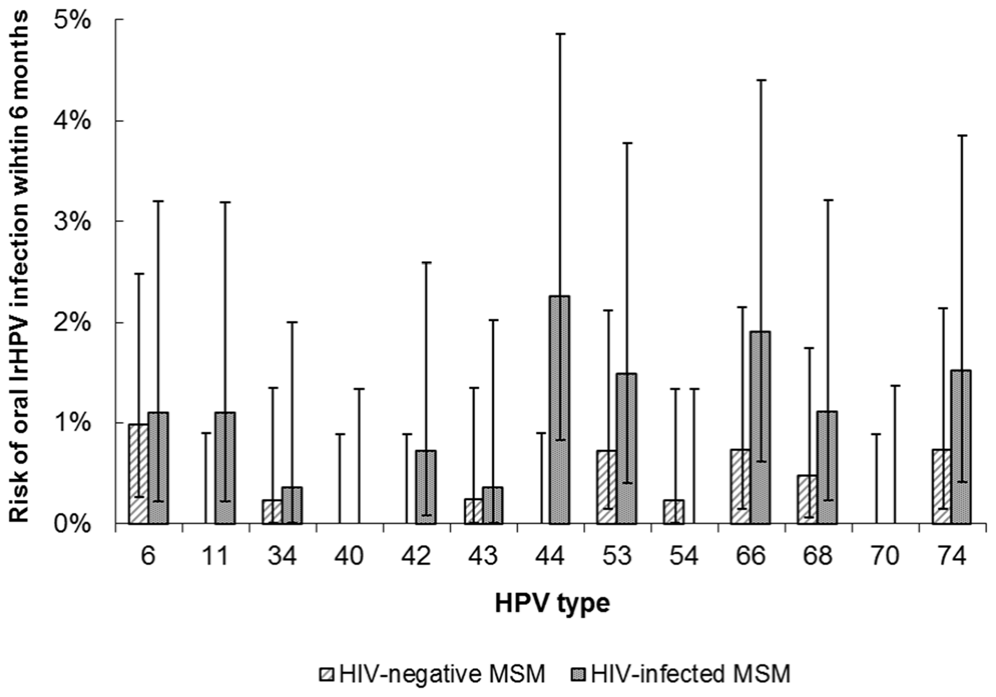
Type-specific 6-month oral low-risk HPV incidence. Type-specific 6-month oral low-risk HPV incidence and 95% confidence intervals in 413 HIV-negative and 276 HIV-infected MSM (H2M study, Amsterdam, 2010–2012). Abbreviations: MSM  =  men who have sex with men; H2M  =  HIV & HPV in MSM; lrHPV  =  low-risk HPV.

Genotyping data were also available for oral low-risk HPV types. Baseline prevalence of low-risk HPV was 7.0% (29/413) in HIV-negative and 20.7% (57/276) in HIV-infected MSM (*P*<0.001). Incidence of ≥1 oral low-risk HPV type at the 6-month visit was observed in 44/689 MSM (6.4%; 95%CI 4.7–8.5): incidence was significantly higher in HIV-infected MSM (27/276; 9.8%; 95%CI 6.5–13.9) than in HIV-negative MSM (17/413; 4.1%; 95%CI 2.4–6.5) (*P* = 0.003). In total, 50 incident low-risk HPV infections were observed (Figure 2).

Of 106 oral low-risk HPV infections present at baseline, 40 (37.7%) persisted and 66 (62.3%) were cleared after 6 months; persistence was observed in 10/32 (31.3%) and 30/74 (40.5%) infections in HIV-negative and HIV-infected MSM, respectively (*P* = 0.3).

### SPF_10_ sequencing results

**Figure 3:**
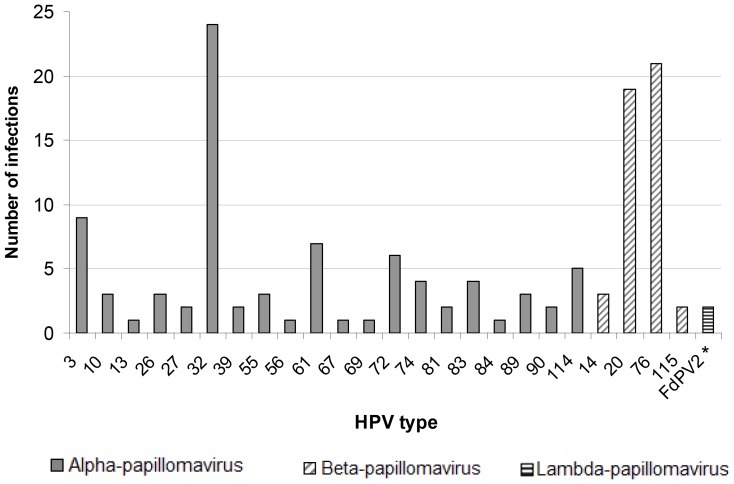
SPF_10_ sequencing results. SPF_10_ sequencing results, showing number of type-specific HPV infections in 131 MSM who had oral samples at baseline or 6-month follow-up that were classified as untypable by LiPA_25_ (H2M study, Amsterdam, 2010–2012). Abbreviations: MSM  =  men who have sex with men; H2M  =  HIV & HPV in MSM. * Felis domesticus Papillomavirus 2.

Ninety of the 106 (85%) untypable baseline samples (PCR-DEIA-positive and LiPA_25_-negative) were successfully analyzed using SPF_10_ sequencing. Additionally, 41 untypable 6-month oral samples from other participants were successfully sequenced. The genotype distribution observed in these 131 samples by use of SPF_10_ sequencing is shown in Figure 3. The most frequently found type was HPV-32 (n = 24). Twenty different Alpha-papillomavirus types, 4 different Beta-papillomavirus types, and one Lambda-papillomavirus type were observed.

From the 6-month untypable samples, 39 samples were from men who also had an untypable sample at baseline. Sequencing of both baseline and 6-month samples was successful in 19 of these 39 participants (48.7%). Type-specific 6-month persistence was observed in 17/19 (89.5%) participants: this concerned HPV types 3, 13, 20 (4 times), 26, 32 (5 times), 74, 76, 83 (2 times), and 114.

We assessed whether we could observe any determinants for HPV-32 and HPV-13 specifically, since these HPV types are known to be associated with focal epithelial hyperplasia [25]. In a total of 31 baseline or 6-month samples, HPV-32 or HPV-13 was identified by use of SPF_10_ sequencing. Increasing number of lifetime male sex partners showed a borderline significant association with HPV-32/HPV-13 infection (OR 1.9; 95%CI 0.6–6.0 for 101–500 partners and OR 3.8; 95%CI 1.3–11.2 for ≥501 partners, compared to ≤100; overall *P* = 0.05). In multivariable analysis adjusting for HIV infection and age, this association remained borderline significant (*P* = 0.07).

## Discussion

In our cohort of MSM, the incidence of oral high-risk HPV was substantial (4.1% in HIV-negative and 14.1% in HIV-infected MSM), and significantly higher among HIV-infected MSM and recent cannabis users. The majority of oral high-risk HPV infections was cleared after 6 months, both in HIV-negative and HIV-infected MSM.

The 6-month oral high-risk HPV incidence observed in our study was high compared to the incidence observed in most other studies, which was not surprising given the high-risk population included in our study. Two studies that took 37 HPV types into account estimated a 12-month oral HPV incidence of 4–10% in men and women aged 18–30 years [18] and a 12-month cumulative incidence of 12% in mostly heterosexual young men [19]. A recent, large study among mostly heterosexual men reported a 12-month oral high-risk HPV incidence of 1.7% [20]. A study among HIV-infected individuals observed an incidence rate of oral high-risk HPV of 9.6 per 1000 person-months [26], which is lower than our findings in HIV-infected MSM. This may be explained in part by differences in study population and laboratory methods used, as our study population exhibited high levels of sexual risk behavior, and our laboratory methods used are very sensitive [23]. In a long follow-up study among pregnant women [27] Louvanto et al. found that HPV-16, which is the predominant type associated with HPV-related head and neck cancer [1], accounted for most incident oral HPV infections compared to other types. In our study population HPV-33 was the most frequent incident HPV type, although type-specific numbers were low.

From studies on cervical infections it is known that persistence of high-risk HPV infection is a very strong risk factor for carcinogenesis. The link between oral high-risk HPV infection and head and neck cancer is much less well established [28]. Although it is very likely, given the similarities with cervical cancer, there is no evidence that persistent high-risk HPV infection is a prerequisite. In our study, 37–38% of oral high-risk and low-risk HPV infections persisted up to 6 months, somewhat lower than the 46% 6-month persistence of any oral HPV infection reported by Beachler et al. [26].

HIV infection was an independent determinant for oral high-risk HPV incidence, which is compatible with the higher oral HPV prevalence among HIV-infected persons reported in previous studies [10], [15] and the generally higher incidence patterns of HPV infections among HIV-infected persons [29]. Whether this increased oral HPV incidence among HIV-infected persons is attributable to HIV-related immunosuppression or to higher sexual risk behavior is unclear. In our multivariable analyses, sexual behavior variables were not significantly associated with oral HPV incidence, although we observed a trend for a higher number of recent oral sex partners. It has been hypothesized that the increased HPV detection in HIV-infected persons can at least partially be explained by more frequent reactivation of latent HPV infection in the context of (local) immunosuppression [30]. We found no association between CD4 cell count and HPV incidence among HIV-infected MSM, but most participants were on combination antiretroviral therapy (cART) and had high CD4 cell counts (117/225 HIV-infected MSM with data on CD4 cell count had more than 500 cells/mm^3^), hence the power of these analyses was limited.

Interestingly, recent cannabis use was associated with oral high-risk HPV incidence. A similar, also borderline significant, association between frequent use of cannabis and oral HPV infection was observed by Pickard et al. [18]. A link between cannabis and HPV-related head and neck cancer has been hypothesized before, for example by Gillison et al. [31], but results across studies are inconsistent [32]. Cannabinoids may play a role in the development of HPV-related head and neck cancer in several ways [31].

To date, the clinical significance of oral HPV infection is unknown, although evidence for a causal role of HPV-16 in subsets of head and neck cancer is accumulating [1]. Our results indicate that both oral high-risk and low-risk Alpha-papillomavirus infection is common among MSM. In addition, a broad range of HPV genotypes can be detected in the oral cavity, which has been previously observed [33]–[35]. Apart from mucosal Alpha-papillomavirus types we observed Beta-papillomavirus types, which are predominantly isolated from the skin and therefore considered cutaneous types. The biological and pathological consequences of these findings require further study. Since the HPV assay that we used is highly sensitive, we cannot rule out contamination from other sites of the body (e.g., hands or facial skin) or from cats, as we observed 2 cases of Papillomavirus 2 associated with Felis domesticus. Although it has been hypothesized that HPV may be transmitted from humans to cats or vice versa [36], these results should be interpreted with caution.

HPV types 13 and 32 have been associated with focal epithelial hyperplasia (Heck's disease) [25], a benign proliferation of the oral mucosa, which is rare in Europe. Previously established risk factors for focal epithelial hyperplasia were related to socio-demographic characteristics, immunological factors, and HIV infection [25]. To our knowledge, this study is the first to describe a possible, albeit non-significant, link between sexual behavior (i.e. lifetime number of sex partners) and oral HPV-13/HPV-32. Since we performed no clinical examination, we have no information on oral lesions indicating symptomatic focal epithelial hyperplasia in these participants.

Strengths of this study include the relatively large sample size, and the detailed information obtained through self-administered questionnaires. The use of oral-rinse-and-gargle samples enabled us to collect cells from the entire oral cavity, which yields more HPV DNA than oral swabs [12], [37]. DNA sequencing of a subset of oral samples confirmed that only few samples (5%) from unique persons that were classified as untypable and successfully sequenced contained HPV DNA of one of the HPV types covered by LiPA_25_.

Our study had some limitations. First, the power of our analyses was limited. Therefore, it was not possible to perform analyses stratified by HIV status. Second, our results cannot be generalized to the general male population, because of our high-risk cohort consisting of HIV-negative and HIV-infected MSM with much (previous) HPV exposure. The effect of behavioral factors could be diluted due to the relatively homogeneous study population with much high-risk behavior, and the effect of kissing and frequency of sex acts was not investigated. Third, in our study we did not differentiate between the use of cannabis with or without the addition of tobacco. Therefore, the observed association between cannabis use and HPV incidence may be (partly) due to the use of tobacco. Fourth, it is quite possible that within the 6-month sampling interval, transient infections were acquired and cleared, or that infections were cleared followed by reinfection. As a consequence, we might have underestimated both incidence and clearance. However, a study evaluating sampling intervals in HIV-infected participants concluded that, although transient oral HPV infections were common, a 6-month sampling interval was appropriate for assessing determinants for oral HPV persistence [38]. On the other hand, since there was only one follow-up visit, it is not determined that all infections classified as “cleared” were truly cleared (e.g. the infection could have remained present under the detection limit of the assay), which could lead to an overestimation of clearance. Fifth, we did not perform clinical examination of the oral cavity, and could therefore not relate individual HPV results to possible oral lesions. Finally, 75/225 (33%) samples untypable by LiPA_25_ could not be successfully sequenced, because of technical difficulties such as the presence of multiple HPV types and the very short PCR fragment (65 bp before nested PCR with elongated primers). This limits the interpretation of our sequencing results.

In conclusion, we observed that incidence of oral high-risk HPV infection in MSM is substantial, and significantly higher in HIV-infected MSM and possibly cannabis users. Nearly two thirds of high-risk HPV infections were cleared within 6 months; we did not identify any independent risk factors for persistence at 6 months. In addition to the known high-risk HPV types, the oral cavity contains a broad range of both mucosal and non-mucosal HPV genotypes. The clinical implications of these HPV types require further research.
